# Successful Implantation of New Coronary Sinus Lead Using Angioplasty for Lead‐Related Vein Occlusion in a CRT‐D Patient

**DOI:** 10.1111/pace.70011

**Published:** 2025-08-21

**Authors:** Ramazan Gunduz, Bekir Serhat Yildiz

**Affiliations:** ^1^ Department of Cardiology Manisa City Hospital Manisa Turkey; ^2^ Ege University Medical Faculty Department of Cardiology Izmir Turkey

**Keywords:** cardiac resynchronization therapy, crush procedure, dislocated coronary sinus lead

## Abstract

This case is a rare occurrence of atrial and coronary sinus (CS) lead dislocation 5 years post cardiac resynchronization therapy‐defibrillator (CRT‐D) implantation, thus necessitating a unique approach. The previous lead‐related vein occlusion was dilated with a balloon using methods typically employed in coronary procedures to address stenosis at the lateral branch of the CS, followed by insertion of a new lead. Materials and techniques used in coronary artery interventional procedures can also be applied in cases of CS vein branch stenosis encountered during CRT‐D implantation. This case represents one of the rare procedures performed in this manner.

**Abbreviations**
CIED: cardiac implantable electronic device
CRT‐D: cardiac resynchronization therapy‐defibrillator
CS: Coronary sinus
ECG: electrocardiogram
ICD: intracardiac defibrillator
LBBB: left bundle branch block
LV: left ventricular
LVEF: left ventricular ejection fraction
NYHA: New York Heart Association
RA: right atrium
RV: right ventricular

## 1 Introduction

Cardiac resynchronization therapy‐defibrillator (CRT‐D) implantations have increased rapidly over the past decade due to improved operator expertise, hardware enhancements, an increase in the number of eligible patients, and expanded indications. Advancements in leads and delivery equipment have resulted in fewer instances of lead dislodgement in modern practice. The most frequent complication associated with the implantation of a CRT‐D is lead dislodgement, typically occurring within 3 months after insertion [1 **]**. Problems following a CRT‐D implantation more frequently arise from the left ventricular (LV) lead, with a reported dislodgement incidence ranging from approximately 4% to 13.6%, whereas dislodgement of the atrial and right ventricular (RV) leads may occur in fewer than 2% of cases [2‐4].

This case is a rare occurrence of atrial and coronary sinus (CS) leads dislocation 5 years after CRT‐D implantation, necessitating a unique approach. The previous lead‐related vein occlusion was dilated with a balloon using methods typically employed in coronary procedures to address stenosis at the lateral branch of the CS, followed by insertion of a new lead.

## 2 Case Report

**FIGURE 1:**
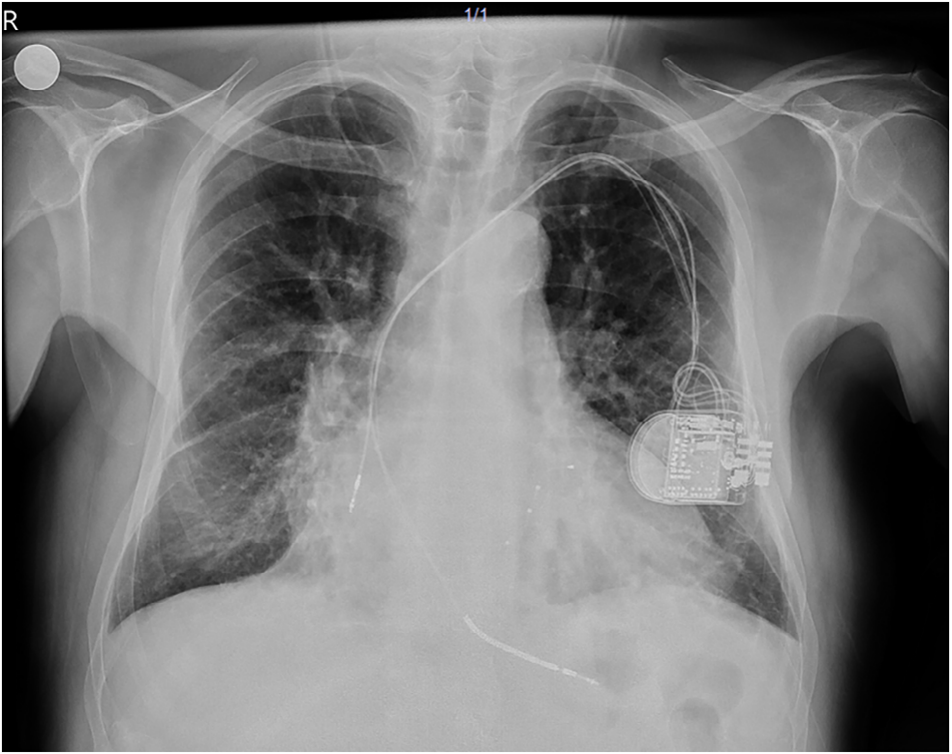
Chest x‐ray before procedure. Antero‐posterior view. [Colour figure can be viewed at wileyonlinelibrary.com]

**FIGURE 2:**
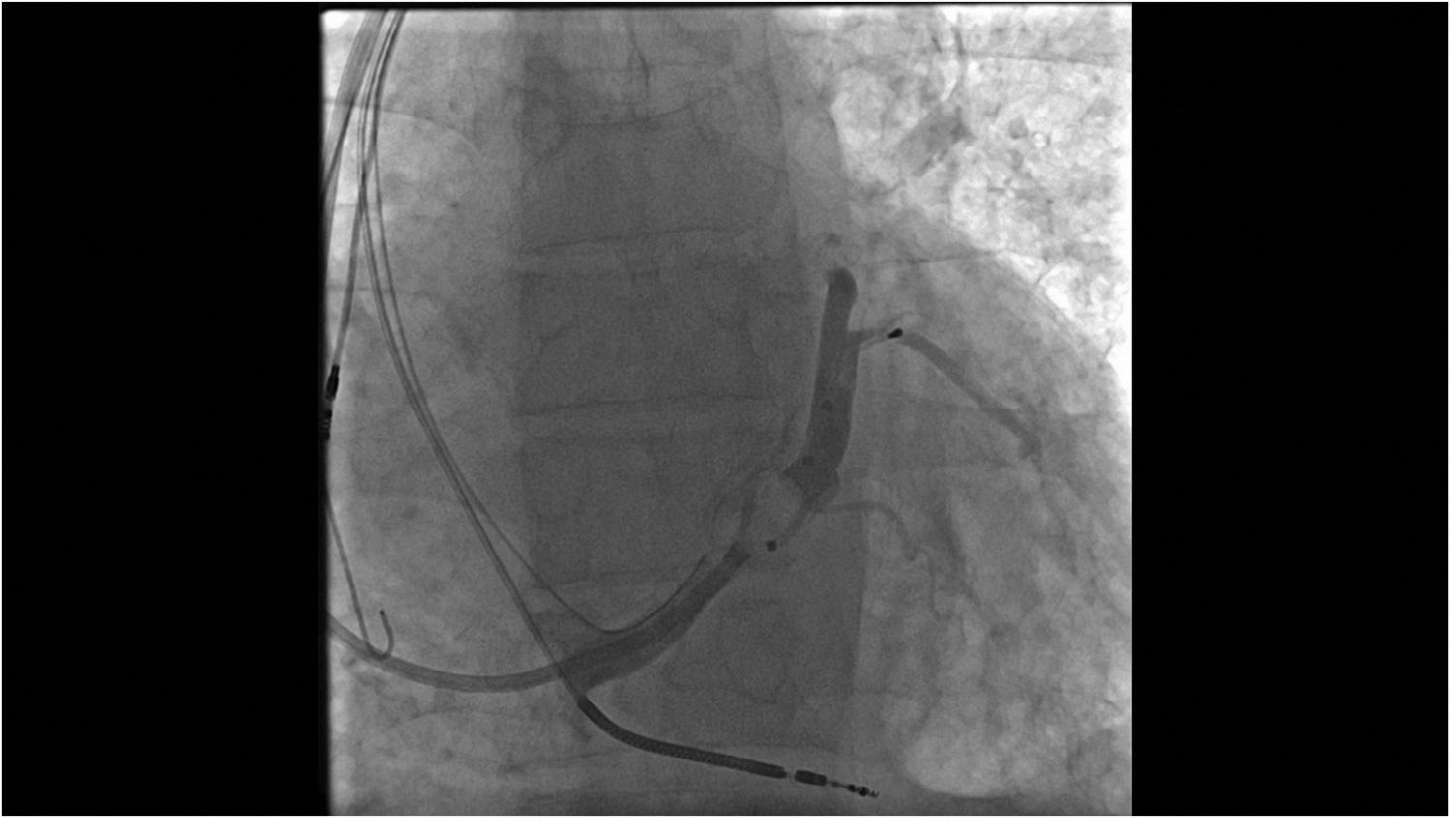
Angiogram of the coronary sinus and its branches with inflated balloon. Antero‐posterior view.

**FIGURE 3:**
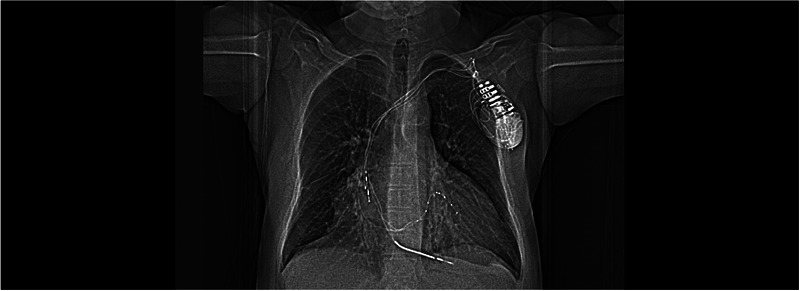
Final chest x‐ray after biventricular device implantation. Antero‐posterior view.

**FIGURE 4:**
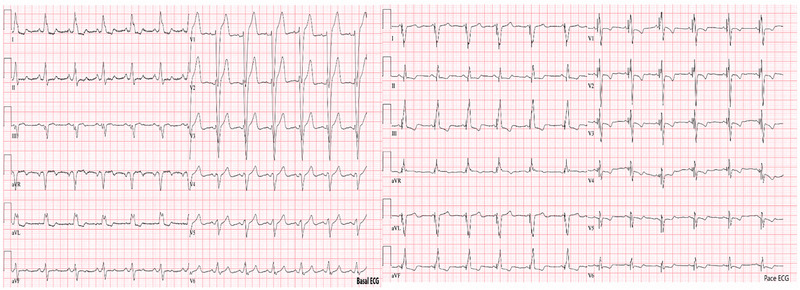
Baseline electrocardiogram and electrocardiogram with biventricular pacing. [Colour figure can be viewed at wileyonlinelibrary.com]

A 65‐year‐old hypertensive man with a history of coronary artery disease and CRT‐D implantation (Medtronic) 5 years earlier was admitted to our hospital with New York Heart Association (NYHA)Class‐III heart failure despite optimal medical therapy. His electrocardiogram (ECG) exhibited a left bundle branch block (LBBB) pattern. Left ventricular ejection fraction (LVEF) was 25%, and the CRT‐D device was operating in ventricular pacing and sensing (VVI) mode. Chest x‐ray and chronogram revealed displacement of the atrial lead and retraction of the LV lead (Figure 1, Video S1). The RV lead parameters were within the normal range (defibrillator lead—pacing threshold: 0.7 V/0.5 ms, R wave: 14.6 mV, and impedance: 460 Ω). No signal could be obtained from the atrial and LV leads. Due to the potential sequelae of only five leads remaining (mechanical, infectious, and anticoagulation), lead extraction was suggested to the patient. However, the patient refused the removal of the leads. Redo of cardiac resynchronization therapy was planned. The procedure was conducted under local anesthesia. During the operation, fibrotic adhesions were observed around the atrial and defibrillator leads in the generator pocket. The CRT‐D generator was replaced with a capsulectomy. At the beginning of the procedure, the atrial and LV leads did not retract manually due to adhesions. The outer delivery sheath was positioned at the ostium of the CS. An Arrow Double‐Lumen Balloon Wedge‐Pressure Catheter was sent through the sheath and inflated inside the CS. A contrasting agent was injected through the balloon to visualize the anatomy of the CS. We observed that the lateral vein, where the distal part of the initially placed CS lead was located, was suitable for the CS lead placement (Video S2). According to the chronogram, there was no other suitable vessel for lead placement (Figure 2). The sheath advanced toward the ostium of the lateral vein. Sequentially, CHOICE Floppy Guidewire (182 cm, 0.014 inch) and CHOICE PT Extra Support (182 cm, 0.014 inch) Guidewire were placed into the CS lead, attempting entry into the lateral vein. Due to the previous CS lead remaining at the ostium of the lateral vein, it created a narrowing, thus preventing entry into the lateral vein. The CHOICE Floppy Guidewire was left inside the lateral vein. The CHOICE PT Extra Support Guidewire was passed through the 2.5 × 15 mm semi‐compliant balloon. The balloon was advanced over the existing wire and maneuvered using the push technique, passing beyond the ostium of the lateral vein. The balloon was inflated to dilate lead‐related vein occlusion (Video S3). Subsequently, the CS lead (ACUITY X4 Straight 4672; Boston Scientific Corporation, USA) was passed through the ostium and positioned within the lateral vein, demonstrating satisfactory electrical parameters (pacing impedance: 700 Ω and pacing threshold: 0.6 V @ 0.5 ms). Subsequently, a new screw‐in INGEVITY MRI 7841 pacing lead (Boston Scientific Corporation, USA) was positioned in the right atrium (RA) appendage (Video S4). The pocket was closed after ensuring satisfactory lead positions and parameters without any complications (Figure 3). After the procedure, the QRS duration was shortened from 160 to 110 ms on the ECG (Figure 4). The patient's NYHA class improved from Class III to Class I at the 1‐month follow‐up.

## 3 Discussion

Placing the LV lead is often considered the most challenging part of CRT implantation. Anomalies in anatomy, such as abnormal positioning of the CS ostium, the presence of venous valves such as the Thebesian valve or Valve of Vieussens, and CS strictures pose challenges during LV lead implantation [5 **]**.

The most prevalent adverse event noted post intracardiac defibrillator (ICD) or CRT‐D implantation is lead dislodgement. Female recipients of defibrillators who are affected by obesity exhibited a notably higher risk of lead dislodgement. This occurrence is linked to device migration, particularly in female patients with obesity [6 **]**. Right atrial and ICD leads exhibited the highest risk of dislodgement at 1.9% compared to that of RV pacemaker and CS leads. The rate of dislodgement or malfunction of CS leads within the first year following implantation is very low [4 **]**.

Dislodgement of the atrial lead may lead to simultaneous sensing of both atrial and ventricular electrograms, inappropriate detection of atrial or ventricular arrhythmias, and loss of atrioventricular synchrony. Dislodgement of LV pacing leads could result in ventricular desynchrony and exacerbation of heart failure. Therefore, prevention, timely diagnosis, and appropriate management of lead dislodgement events are crucial for the management of cardiac implantable electronic devices (CIED). Apart from this, one of the most important complications is the risk of infection and thrombosis due to dislodged leads left in place. Normally, it is more appropriate to remove these leads before implanting a new one. In this patient, we initially recommended lead extraction, but the patient refused [7 **]**.

Typically, the standard approach for initial LV placement is guided by anatomical considerations. The lead tip is placed proximate to the basal region of the lateral wall at a stable location exhibiting satisfactory pacing parameters while ensuring avoidance of left phrenic nerve capture.

Challenges we may encounter during CRT implantation or upgrade include variations in venous anatomy or stenosis. Performing percutaneous venoplasty to manage venous stenosis prior to lead implantation may facilitate a successful CRT upgrade [8 **]**. If this narrowing occurs at the ostium or branches of the CS, the procedure becomes more challenging during CRT implantation. Another option is conduction system pacing. This is an emerging technique that may demonstrate beneficial effects on CRT regarding improvements in ejection fraction, procedural complexity, impact on survival, and hospitalizations due to heart failure. However, there was no conduction pacing system available in the angiography laboratory at the time we performed this procedure [9 **]**.

In this case, the LV lead was positioned in the lateral vein ostium, which was the only suitable branch, but it caused narrowing. We were able to place a second lead by utilizing an innovative technique similar to balloon predilatation used in coronary stenosis to widen the ostium of the lateral vein.

## 4 Conclusion

Materials and techniques used in coronary artery interventional procedures can also be applied in cases of CS vein branch stenosis encountered during CRT‐D implantation. This case represents one of the rare procedures performed in this manner.

## Ethics Statement

The study complied with the Declaration of Helsinki, and informed consent was obtained from the participant.

## Consent

The patient consent statement was taken.

## Conflicts of Interest

The authors declare no conflicts of interest.

## Statement on the Authenticity of Figures and Videos

The authors attest that all submitted figures and videos have been generated by them, affirming that the images are entirely original, without duplication, and have not been previously published either in full or in part.

## Supporting information




**Video 1**: Dislodged leads before procedure.

**Video 2**: Coronary sinus angiogram. Lateral vein ostial stenosis.

**Video 3**: Balloon inflation in narrowed lateral vein.

**Video 4**: Final leads location after procedure.

## Data Availability

Data sharing is not applicable to this article as no datasets were generated or analyzed during the current study.
